# Supplementary material for: Efficient spin accumulation carried by slow relaxons in chiral tellurium
Source: Nat Commun. 2025 Apr 30;16:4056. doi: 10.1038/s41467-025-59143-0 (PMC12044024; doi:10.1038/s41467-025-59143-0)
Supplement: Supplementary file 1 — Supplementary Information [file 41467_2025_59143_MOESM1_ESM.pdf]

Supplementary Information for

Efficient spin accumulation carried by slow relaxons in chiral tellurium

Evgenii Barts, Karma Tenzin, and Jagoda Sławińska

Zernike Institute for Advanced Materials, University of Groningen,

Nijenborgh 3, 9747 AG Groningen, The Netherlands

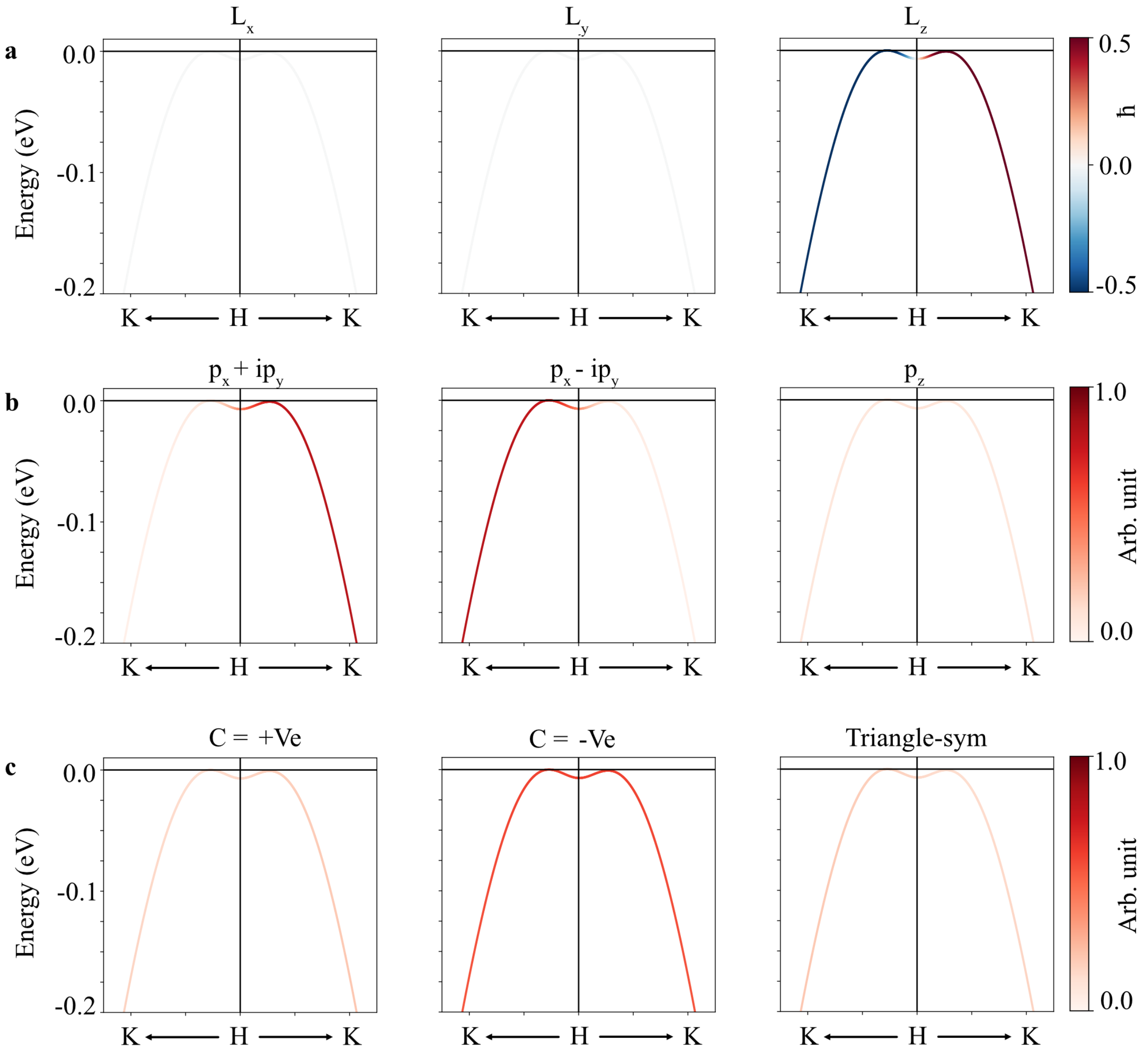

Supplementary figure 1: **Calculated valence band structure along the KHK line in the Brillouin zone in the left-handed Te.** Color shows **a** expectation value of the onsite orbital momentum, **b** wavefunction projection on atomic p-orbitals, and **c** projection on three sublattice states, where $\pm$ chirality corresponds to the state $(1, e^{\pm 2\pi i/3}, e^{\mp 2\pi i/3})/\sqrt{3}$ and the symmetric achiral state is $(1, 1, 1)/\sqrt{3}$.

Supplementary note 1

In Supplementary Fig. 1 we show calculated valence band structure along the KHK line in the Brillouin zone. Specifically, we separately identify chiral properties of the wavefunctions close to the low-doping hole pocket at the H point by projecting them onto different orbital and sublattice states. Panel **a** shows that the states are strongly polarized orbital momentum L_z. It is supported by panel **b**, which confirms that the right part of the band primarily are $p_x + ip_y$ states, whereas the left part are $p_x - ip_y$, since the p-orbital states are explicitly given in terms of $|L, L_z\rangle$ as:

$$\begin{aligned} |1,1\rangle &= -\frac{1}{\sqrt{2}}(p_x + ip_y), \\ |1,0\rangle &= p_z, \\ |1,-1\rangle &= \frac{1}{\sqrt{2}}(p_x - ip_y). \end{aligned} \tag{S1}$$

To obtain the orbital polarization separately from spin polarization, we calculate the expectation values of $\hat{L}_z = |1,1\rangle\langle 1,1| - |1,-1\rangle\langle 1,-1|$, which leads to the expression for orbital polarization in the main text. Since the strong SOC in Te ($\lambda \approx 0.45$ eV) splits the p-orbital states into a quadruplet and a doublet ($|J, J_z\rangle$ with $J = 3/2$ and $J = 1/2$) the partial spin and orbital polarization of states is likely a result of the mixing of oppositely-polarized states within the higher-energy quadruplet:

$$\begin{aligned} |\frac{3}{2},\frac{3}{2}\rangle &= -\frac{1}{\sqrt{2}}(p_x + ip_y)\uparrow, \\ |\frac{3}{2},\frac{1}{2}\rangle &= -\sqrt{\frac{1}{6}}(p_x + ip_y)\downarrow + \sqrt{\frac{2}{3}}p_z\uparrow, \\ |\frac{3}{2},-\frac{1}{2}\rangle &= \sqrt{\frac{2}{3}}p_z\downarrow + \sqrt{\frac{1}{6}}(p_x - ip_y)\uparrow, \\ |\frac{3}{2},-\frac{3}{2}\rangle &= \frac{1}{\sqrt{2}}(p_x - ip_y)\downarrow, \\ |\frac{1}{2},\frac{1}{2}\rangle &= -\frac{1}{\sqrt{3}}(p_x + ip_y)\downarrow - \frac{1}{\sqrt{3}}p_z\uparrow, \\ |\frac{1}{2},-\frac{1}{2}\rangle &= \frac{1}{\sqrt{3}}p_z\downarrow - \frac{1}{\sqrt{3}}(p_x - ip_y)\uparrow. \end{aligned} \tag{S2}$$

Note that these atomic wavefunctions naturally serve as the basis states in the PAOFLOW package, allowing us to calculate both spin and onsite orbital components by using $\hat{J}_z = \left(\hat{S}_z + \hat{L}_z\right) = \sum_{J,J_z} J_z\, |J, J_z\rangle\langle J, J_z|$.

Panel **c** shows that left-handed Te also imposes significant polarization in sublattice degrees of freedom. When the three sublattice states are written in the chiral basis, wherein $\pm$ chirality corresponds to the state $(1, e^{\pm 2\pi i/3}, e^{\mp 2\pi i/3})/\sqrt{3}$ and symmetric achiral state to $(1,1,1)/\sqrt{3}$, all the states close to the H point belong to the states with negative sublattice chirality.
